# Increases in cytosolic Ca^2+^ induce dynamin- and calcineurin-dependent internalisation of CFTR

**DOI:** 10.1007/s00018-018-2989-3

**Published:** 2018-12-13

**Authors:** Waseema Patel, Patrick J. Moore, M. Flori Sassano, Miquéias Lopes-Pacheco, Andrei A. Aleksandrov, Margarida D. Amaral, Robert Tarran, Michael A. Gray

**Affiliations:** 1 Institute for Cell and Molecular Biosciences, Newcastle University, Newcastle upon Tyne, NE2 4HH UK; 2 Marsico Lung Institute, The University of North Carolina at Chapel Hill, Chapel Hill, NC USA; 3 Faculty of Sciences, BioISI-Biosystems and Integrative Sciences Institute, University of Lisboa, Lisbon, Portugal; 4 Department of Biochemistry and Biophysics, Cystic Fibrosis Research and Treatment Center, The University of North Carolina at Chapel Hill, Chapel Hill, NC USA; 5 Department of Cell Biology and Physiology, Cystic Fibrosis Research and Treatment Center, The University of North Carolina at Chapel Hill, Chapel Hill, NC USA

**Keywords:** CFTR, Calcium, Cigarette smoke, Calcineurin

## Abstract

The cystic fibrosis transmembrane conductance regulator (CFTR) is a cAMP-regulated, apical anion channel that regulates ion and fluid transport in many epithelia including the airways. We have previously shown that cigarette smoke (CS) exposure to airway epithelia causes a reduction in plasma membrane CFTR expression which correlated with a decrease in airway surface hydration. The effect of CS on CFTR was dependent on an increase in cytosolic Ca^2+^. However, the underlying mechanism for this Ca^2+^-dependent, internalisation of CFTR is unknown. To gain a better understanding of the effect of Ca^2+^ on CFTR, we performed whole cell current recordings to study the temporal effect of raising cytosolic Ca^2+^ on CFTR function. We show that an increase in cytosolic Ca^2+^ induced a time-dependent reduction in whole cell CFTR conductance, which was paralleled by a loss of cell surface CFTR expression, as measured by confocal and widefield fluorescence microscopy. The decrease in CFTR conductance and cell surface expression were both dynamin-dependent. Single channel reconstitution studies showed that raising cytosolic Ca^2+^ per se had no direct effect on CFTR. In fact, the loss of CFTR plasma membrane activity correlated with activation of calcineurin, a Ca^2+^-dependent phosphatase, suggesting that dephosphorylation of CFTR was linked to the loss of surface expression. In support of this, the calcineurin inhibitor, cyclosporin A, prevented the Ca^2+^-induced decrease in cell surface CFTR. These results provide a hitherto unrecognised role for cytosolic Ca^2+^ in modulating the residency of CFTR at the plasma membrane through a dynamin- and calcineurin-dependent mechanism.

**Electronic supplementary material:**

The online version of this article (10.1007/s00018-018-2989-3) contains supplementary material, which is available to authorized users.

## Introduction

CFTR is a cAMP- and ATP-regulated anion channel whose function is critical for transepithelial anion and fluid secretion in multiple organs, including the conducting airways, intestines, reproductive tracts, and kidneys [1, 2]. The importance of the channel is underscored by the hereditary disease cystic fibrosis (CF), where loss of anion permeability through CFTR has major detrimental effects in the lungs, gastrointestinal and reproductive tracts. Critically, the deterioration of lung function is a major determinant of morbidity in CF [1]. We, and others, have recently linked the loss of CFTR function to the development and progression of the chronic bronchitis (CB) form of chronic obstructive pulmonary disease (COPD) [3, 4]. COPD is often caused by smoking and is characterised by airflow obstruction, which is clinically seen as a manifestation of either CB, emphysema, or a combination of both. CB is defined as a chronic cough that persists for greater than 2 months. Importantly, CB bears many phenotypic similarities to CF, such as increased mucus stasis and airway inflammation [5].

CFTR itself is composed of two membrane spanning domains, each of which contains six transmembrane regions and two nucleotide binding domains (NBDs) which bind ATP. Unique to CFTR is the regulatory ‘R’ domain. The functional properties of CFTR have been classically characterised using electrophysiology; CFTR has a single channel conductance of 8–10 pS and a linear current–voltage relationship [6, 7]. CFTR is activated following PKA phosphorylation of the R domain, together with ATP binding and dimerisation of the two NBDs, a process which is enhanced by PKC phosphorylation [8–11]. Conversely, CFTR is inactivated by dephosphorylation of the R domain via several protein phosphatases, including protein phosphatase (PP) 2A, PP2B (also known as calcineurin) and PP2C [12–15]. In addition to phosphorylation-dependent regulation of CFTR activity, the whole cell CFTR conductance is also dependent on the number of CFTR channels at the cell surface. The trafficking of CFTR to the plasma membrane is a highly regulated process, with the protein passing through multiple quality control steps within the endoplasmic reticulum (ER) and Golgi compartments before it reaches the cell surface [16–18]. CFTR is conventionally internalised via a dynamin- and clathrin-mediated process [19]. The majority of internalised CFTR is routed to recycling endosomes, allowing for a large repository of functional CFTR protein that remains readily available for transport back to the membrane, if required. Alternatively, the protein can be routed to the lysosomes for degradation [20, 21].

We have previously demonstrated that acute exposure of fully differentiated primary airway epithelial cultures to CS caused a time-dependent loss of CFTR from the plasma membrane, which correlated with a reduction in airway surface liquid (ASL) volume (i.e. dehydration) [4, 22]. Furthermore, the loss of CFTR function was shown to be dependent upon a CS-induced increase in cytosolic Ca^2+^. This Ca^2+^ emanated from intracellular stores (probably lysosomal) and did not involve Ca^2+^ influx across the plasma membrane, consistent with other studies showing that CS and its constituents cause increases in cytosolic Ca^2+^ [22–24]. Importantly, we showed that preventing a CS-induced increase in cytosolic Ca^2+^ prevented CFTR internalisation [22], thereby linking a change in cytosolic Ca^2+^ to the loss of CFTR function. Within airway epithelia, Ca^2+^ regulates processes including Cl^−^ secretion through Ca^2+^-activated Cl^−^ channels, mucus secretion, and ciliary beat frequency [25–28]. However, whether an increase in cytosolic Ca^2+^ through mechanisms other than via exposure to CS could modulate the residency of CFTR at the plasma membrane has not been previously investigated. To investigate this possibility, we have measured the temporal effect of raising cytosolic Ca^2+^ by a range of Ca^2+^ agonists on CFTR activity using real-time measurements of single channel and whole cell CFTR current recordings, as well the temporal effect of raising Ca^2+^ on cell surface expression as determined by confocal and widefield fluorescence microscopy.

## Materials and methods

### Cell culture

Human embryonic kidney (HEK) 293T cells for patch clamp experiments were cultured in Dulbecco’s modified Eagle’s medium (DMEM) supplemented with 2 mM l-glutamine, 1% non-essential amino acids, 10% foetal bovine serum (FBS), 100 U ml^−1^ penicillin and 100 µg ml^−1^ streptomycin. HEK293T cells used for imaging experiments were cultured in DMEM supplemented with 10% FBS, 100 U ml^−1^ penicillin and 100 µg ml^−1^ streptomycin. CF bronchial epithelial (CFBE41o^−^) cells stably expressing the mCherry-Flag-WT-CFTR construct were cultured in DMEM supplemented with 10% FBS, 10 µg ml^−1^ blasticidin and 2 µg ml^−1^ puromycin. This construct contains a reporter under the control of a Tet-ON promoter [29]. Primary human bronchial epithelial cells (HBECs) were obtained by the University of North Carolina’s Cystic Fibrosis Center Tissue Core in a procedure approved by the University of North Carolina Institutional Committee for the Protection of the Rights of Human Subjects. Cells were grown using a base media of DMEM-H:LHC (50:50) with additives as detailed previously [30]. Cells were grown under air–liquid interface until fully differentiated. All cells were grown at 37 °C in a humidified 5% CO_2_ atmosphere.

### Plasmids and transfection

HEK293T cells were transiently transfected with the bicistronic pIRES2-EGFP-CFTR vector to co-express wild-type CFTR and enhanced green fluorescent protein (GFP) for patch clamp experiments [31]. Briefly, DNA was pre-complexed with Lipofectamine 2000 (Invitrogen) and Opti-MEM with GlutaMAX (Invitrogen) for 15 min at room temperature. DNA was then diluted in culture media to 1 µg ml^−1^ and added to cells. Following 6 h incubation at 37 °C, cells were left to incubate overnight in Opti-MEM with 10% FBS after which cells were transferred back to culture media. Cells were studied 48–72 h post transfection at ~ 50% confluency. For imaging experiments, cells were transfected with 1 µg wild-type CFTR with a GFP tag on the N terminus of the channel, CFTR containing an external HA-tag [20, 22] or a dominant negative dynamin mutant, dynamin K44A (Addgene plasmid #34683). DNA and Lipofectamine 2000 (Invitrogen) were diluted in Opti-MEM and the mixtures were incubated for 5 min at room temperature. Following the incubation, the diluted DNA was mixed with the diluted Lipofectamine 2000 and incubated for 15 min at room temperature. The cells were transferred to growth media without antibiotics and the transfection mix was added dropwise. Cells were incubated for 4 h at 37 °C after which the transfection mix was aspirated and the cells were transferred back to culture media. Cells were used 48 h post transfection at 60% confluency.

### Electrophysiology

Whole cell recordings were carried out on single cells and all experiments were conducted at room temperature as previously described [32, 33]. Pipettes were pulled from borosilicate glass capillaries (GC120F; Harvard Apparatus, Kent) using the P-87 Sutter Flaming/Brown micropipette puller. All pipettes had resistances between 3 and 5 MΩ after fire-polishing; the pipette solution was filtered through a 0.2-µm filter before use. The pipette solution contained in mM; 120 CsCl, 2 MgCl_2_, 0.2 EGTA, 10 HEPES and 1 Na_2_ATP, set to pH 7.2 with CsOH. Pipette solution osmolarity was typically 260 mOsm. Pipette solution contained low EGTA to prevent a buffering of increases in cytosolic Ca^2+^ [34, 35]. The bath solution contained in mM; 130 NaCl, 5 KCl, 1 CaCl_2_, 1 MgCl_2_, 10 HEPES, 10 glucose and 20 mannitol, set to pH 7.4 with HCl. For nominally Ca^2+^-free bath solution, CaCl_2_ was omitted and 2 mM MgCl_2_ was added to maintain the Cl^−^ gradient. Bath solution osmolarity was typically 310 mOsm. Experiments were carried out using an Axopatch 200B (Molecular Devices Inc) patch clamp amplifier and data were captured using pClamp10 software. The reference electrode was a silver/silver chloride wire connected to a 150-mM NaCl agar bridge. Cells were held at 0 mV and voltage steps were applied between ± 100 mV in 20 mV increments. Each voltage step lasted 250 ms and there was a 1-s interval between subsequent steps. Data were filtered at 1 kHz and sampled at 2 kHz with a four-pole Bessel filter. The average current obtained between a 100-ms period starting 80 ms into the voltage step was used to construct current–voltage (*I*–*V*) plots. Liquid junction potentials were corrected for and applied to membrane potentials. The Axopatch 200B amplifier was used to compensate for series resistance; 70% compensation was used for all whole cell recordings. The input capacitance of cells was measured before each experiment and compensated for. Slope conductance was calculated by fitting a linear regression to each *I*–*V* plot. Single cell slope conductance was divided by cell capacitance (pF) to normalise data to cell size and is expressed as nS/pF.

### Lipid bilayer-based single channel recording

Single channel CFTR activity was measured using purified CFTR expressed in planar lipid bilayers as previously described [36, 37]. Channel activity was recorded after exposure to Ca^2+^-free conditions (in mM; 5 MgATP, 3 Mg^2+^, 1 EGTA and 300 Tris–HCl, pH 7.2) and a Ca^2+^-containing solution (in mM; 4.5 MgATP, 0.32 CaATP, 0.18 ATP, 3.5 Mg^2+^, 1 CaEGTA, 0.18 Ca^2+^) on the cytosolic face of CFTR. An all points histogram by multipeak Gaussian was fitted to the data and single channel conductance was calculated using the distance between peaks on the all points histogram. Channel open probability was calculated using the ratio of the area under the peak when the channel was open compared to the total area.

### Confocal microscopy

HEK293T cells were imaged using a Leica TCS SP8 confocal laser scanning microscope. Images were captured using a 63 × 1.3 NA oil immersion lens, with a bidirectional scan frequency of 700 Hz and a pinhole of 1 airy unit. GFP was excited with the 488 nm line of an argon laser. Images were captured using the Leica Application Suite: Advanced Fluorescence (LAS AF) software.

Images were analysed offline using ImageJ by manually selecting 6 regions of equal size from the plasma membrane and 6 regions from the intracellular space. Brightfield images were used to select regions of interest from the plasma membrane where fluorescence had been lost due to internalisation of CFTR. Any membranes connecting two adjacent cells were excluded from analysis. The average intensity of the six regions was then determined for each cell. The average values of all the cells from either vehicle-treated and air-exposed cells were collected and were taken as one; all other treatments were normalised as previously described [22].

### Widefield epifluorescence microscopy

CFBE41o^−^ mCherry-Flag-WT-CFTR cells were seeded onto 384-well plates (2.5 × 10^3^ cells/well) using a Multidrop Combi peristaltic dispenser (Thermo Scientific). The next day, CFTR expression was induced with antibiotic-free medium supplemented with 1 µg ml^−1^ doxycycline. 24 h after induction of CFTR expression, cells were treated with DMSO, thapsigargin or ionomycin for up 2 h. Thereafter, extracellular Flag-tags were immunostained in non-permeabilised cells as previously described [29]. Cell imaging was performed with an automated widefield epifluorescence microscopy (Leica DMI6000B), and image analysis was performed using a previously established automatic pipeline in CellProfiler software [29].

### ASL height measurements

HBECs were washed with PBS for 1 h to remove excess mucus before all experiments. Cells were then loaded with 1 mg ml^−1^ tetramethylrhodamine conjugated to 10 kDa dextran. Before imaging, the cultures were transferred to Ringer’s solution and 100 µl perfluorocarbon (PFC, FC-770; Acros Organics) was added mucosally to prevent evaporation of the ASL. Compounds added apically in these experiments were suspended as a dry powder in PFC and sonicated for 10 min.

ASL was imaged using a XZ scan on a confocal laser scanning microscope (Leica SP5) with a 63× glycerol immersion lens and the 561 nm laser. Images were taken at 12 predefined points on the transwell. Images were analysed offline using ImageJ software.

### CFTR surface labelling and on cell Western blot

For surface labeling of CFTR, HEK293T cells were transfected with HA-CFTR using Lipofectamine 2000 and plated on 35 mm glass bottom cell culture dishes. 48 h later, cultures were incubated for 1 h at 4 °C, with a blocking solution (1% BSA/1% NGS) and another 1 h at 4 °C with mouse anti-HA antibody (ab) conjugated to Alexa-488 (1:1000; Biolegend) or rabbit anti-calnexin ab (Cell Signalling Technology) in blocking solution. Cultures were washed three times at 4 °C in ice-cold PBS to remove excess ab, placed in HEK293T media warmed up to 37 °C and exposed to vehicle or thapsigargin. After 1 h incubation at 37 °C with 5% CO_2_, cultures were fixed in 4% PFA and permeabilised with 1% Triton-X. DAPI was added for 30 min for nuclear staining and cells were washed three more times with PBS and then imaged using a Leica SP8 confocal microscope with a 63 × 1.3 NA oil immersion lens. Fluorophores were excited with 405, 488 and 647 nm laser lines and images were collected using sequential scanning to avoid fluorescent bleed through. To determine the percentage co-localisation between CFTR and calnexin, images were overlaid, and the mean Pearson’s correlation coefficient, as an indicator of colocalisation, was quantified using the LAS-AF software (Leica). The following calculation was automatically used by the LAS AF software to determine the percentage colocalization between colocalized areas and background within the ROI, with single cells being chosen as an ROI. Percent colocalisation = colocalisation area/area foreground, where area foreground = image area/area background.

For on-cell Western blot, HEK293T cells were transiently transfected with a HA-CFTR and/or the dynamin K44A plasmid using Lipofectamine 2000 as per the manufacturers’ instructions (ThermoFisher Scientific). After overnight incubation, transfected cells were plated in 384-well plates (10,000 cells/well) and studied 24 h later. Cultures were treated with thapsigargin or vehicle diluted in HEK293T media at 0, 15, 30 and 60 min. The media was then replaced with standard Ringer’s solution (50 μl/well). To halt the experiment, cultures were placed in ice-cold media at 4 °C, followed by a 1-h incubation at 4 °C, with a blocking solution (10% FBS and 1% BSA). This solution was replaced with antibody solution (1:500 mouse anti-HA antibody conjugated to Alexa-488, HA-488 antibody, from Biolegend) and incubated for 1 h at 4 °C. Cultures were then fixed using 4% PFA and rinsed 3× with cold PBS. Fluorescence (488 nm) was measured using a Cytation5 cell imaging multi-mode reader (Biotek). Cultures were then stained using DAPI nuclear dye to give an indicator of cell number. Background fluorescence was determined by exposing non-transfected/antibody-exposed HEK293T cells to HA-488 antibody and each well was background-subtracted. Images were analysed offline using ImageJ software; regions of interest were selected using the threshold function and data were then background subtracted against non-transfected wells.

### Cigarette smoke exposure

Whole cigarette smoke was generated manually using 3R4F reference cigarettes from the University of Kentucky (Lexington, Kentucky). Cells were exposed to 13 × 35 ml puffs of 2 s duration every 30 s, which was the equivalent of one research cigarette. The particulate fraction of smoke is highly autofluorescent and was removed by placing a Cambridge filter pad in the output line. Before exposure, cells were rinsed in pyruvate Ringer’s solution and left to incubate in Ringer’s for 5 min at 37 °C. Following incubation, the excess Ringer’s solution was tipped off, and cells were then exposed to smoke or room air in dedicated chambers for each. Following smoke exposure, cells were placed back into culture media and incubated for the times indicated before fixation.

### Measurement of intracellular Ca^2+^

HEK293T were loaded with Fura-2 acetoxymethylester (AM; 5 µM) to measure changes in cytosolic Ca^2+^; all experiments were carried out at room temperature. Cells were subsequently placed in chambers with entry and exit ports for CS or air and manually exposed to either one filtered reference cigarette or the equivalent of room air as described above. Single cells were selected as regions of interest and images were acquired every 30 s with HCImageLive software (Hamamatsu). Experiments were carried out using an inverted microscope (Nikon, USA) and were viewed under an oil immersion lens (Nikon, Fluor 40, numerical aperture 1.3). The microscope was equipped with a 200-W metal halide lamp and shutter that contained 340 nm and 380 nm bandpass filters; emission at 510 nm was measured using an Orca CCD camera (Hamamatsu). The emission ratio of the 340 nm and 380 nm wavelengths was taken to be a read out of cytosolic Ca^2+^. Counts were corrected for background fluorescence. Changes in cytosolic Ca^2+^ are expressed as maximum changes in the 340/380 ratio induced by each agent from the resting ratio. Area under the curve (AUC) was also chosen to indicate changes in Ca^2+^ as this gives an estimation of the magnitude of any changes as well as any subsequent recovery.

### Measurement of calcineurin phosphatase activity

Calcineurin phosphatase activity was assayed using a colourimetric test from Enzo life sciences kit as per manufacturer’s instructions. HEK293T cells were seeded onto 60 mm tissue culture dishes and treated 24 h later. Total phosphatase activity was measured in 3 µg of sample.

### Statistics

Data are presented as mean ± SEM. Analyses were carried out on Graphpad Prism v5. Significance was tested using Friedman test with Dunn’s post-test for patch clamp experiments, Kruskal–Wallis with Dunn’s post-test or a one sample *t* test for all other experiments, as appropriate. A *p* value < 0.05 was considered statistically significant.

## Results

### An increase in cytosolic Ca^2+^ causes a reduction in CFTR-mediated conductance

**Fig. 1:**
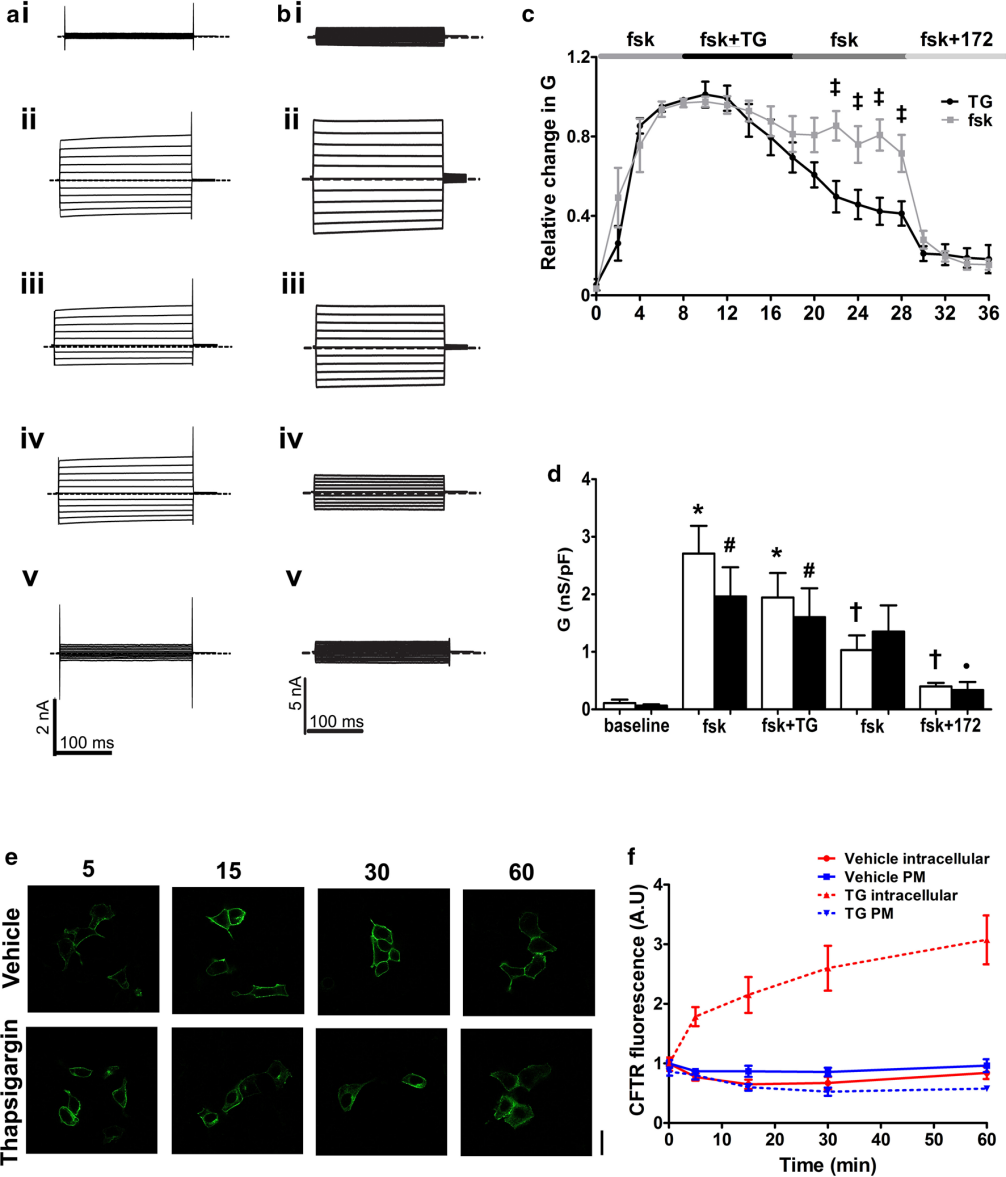
Thapsigargin causes a decrease in CFTR-mediated conductance. **a** Representative fast whole cell current traces obtained by holding the membrane potential at 0 mV and applying voltage steps between ± 100 mV in 20 mV increments under (**i**) basal conditions and after (**ii**) 8 min, (**iii**) 18 min and (**iv**) 28 min perfusion with forskolin (fsk; 5 µM) and (**v**) fsk and CFTR_inh_-172 (10 µM). **b** Representative fast whole cell current traces obtained as in **a** under (**i**) basal conditions (**ii**) after 8 min perfusion with forskolin (fsk; 5 µM) (**iii**) 10 min perfusion with fsk and thapsigargin (TG; 200 nM) (**iv**) 10 min perfusion with fsk alone and (**v**) 8 min perfusion with fsk and CFTR_inh_-172. Dashed line indicates the zero current level. **c** Mean changes in conductance (*G*) plotted relative to the maximum current reached at + 100 mV when cells were perfused with fsk or after TG exposure as indicated. ^ǂ^*p* < 0.05 when compared to equivalent timepoint in cells exposed to forskolin only. **d** Changes in conductance under the conditions indicated. Conductance was normalised to cell size. Data was taken from timepoints indicated in **a** and **b**. Data are mean ± SEM (*n* = 4–10 cells). Open bars represent cells exposed to thapsigargin, closed bars represent cells exposed to forskolin only. *p < 0.05 when compared to baseline in fsk only treated cells. ^†^*p* < 0.05 when compared to maximal forskolin stimulated conductance at 8 min in fsk only treated cells. ^#^*p* < 0.05 when compared to baseline in fsk + TG treated cells ^•^*p* < 0.05 when compared to maximal forskolin stimulated conductance at 8 min in cells treated with TG. **e** Representative images showing the effect of vehicle and thapsigargin on CFTR at timepoints indicated. **f** Changes in intracellular and membrane fluorescence. A single exponential was fitted to the data to give a half-life of 11.9 min and a *τ* of 17.2. Data are mean ± SEM (*n* = 63–86 cells from 6 coverslips). Scale bar represents 25 µm

To investigate the effect of raising cytosolic Ca^2+^ on CFTR channel activity, we first studied the effect of altering cytosolic Ca^2+^ on whole cell CFTR Cl^−^ currents in CFTR-transfected HEK293T cells. To activate CFTR cells were exposed to forskolin, which caused a time-dependent increase in whole cell conductance (Fig. 1a, c; grey trace). The increase in conductance reached a plateau within 8–10 min and then remained relatively stable thereafter, with 69.4 ± 10% of the forskolin-activated conductance still present 20 min after the conductance reached a maximum. At this point, addition of the selective CFTR blocker, CFTR_inh_-172 (10 µM) [38], caused a significant inhibition of whole cell conductance, confirming that CFTR was responsible for this current (Fig.1c; grey trace).

Our previous experiments showed that CS induced Ca^2+^-dependent inhibition of CFTR [22]. To try and reproduce this effect during patch clamp recordings, (which could not employ CS), we instead exposed cells to the sarcoendoplasmic reticulum Ca^2+^ ATPase (SERCA) pump inhibitor, thapsigargin, which causes release of Ca^2+^ from the ER (Fig. S1) [39]. Note that for these patch clamp experiments the cytosolic (pipette) solution contained a low concentration of the Ca^2+^ buffer, EGTA (0.2 mM), which allows Ca^2+^ to increase upon agonist stimulation [34, 35]. Figure 1b shows that in cells in which CFTR had been maximally activated by forskolin, exposure to thapsigargin (200 nM) for 10 min led to a markedly accelerated loss of the whole cell CFTR conductance, compared to control cells (Fig. 1b, c; black trace). The CFTR currents continued to decline even after washout of thapsigargin, and only 40.0 ± 6.5% of the CFTR conductance remained 20 min after the initial exposure to thapsigargin (Fig. 1c; black trace). However, thapsigargin did not totally abolish all CFTR-mediated conductance at this point, as some CFTR_inh_-172 sensitive conductance remained (Fig. 1c, d).

To characterise the effect of an increase in cytosolic Ca^2+^ on CFTR trafficking, we used confocal microscopy to assess changes in the cellular location of CFTR. These experiments revealed CFTR was lost from the plasma membrane after exposure to thapsigargin with a half-life of 11.9 min and a *τ* of 17.2. Intracellular fluorescence increased consistent with an increase in cytosolic Ca^2+^ inducing the internalisation of CFTR. However, when cells were exposed to vehicle, there was little change in either plasma membrane or intracellular fluorescence (Fig. 1e, f).

### Ca^2+^ release from cellular stores is sufficient to cause a reduction in CFTR-mediated conductance without affecting the single channel kinetics of CFTR

**Fig. 2:**
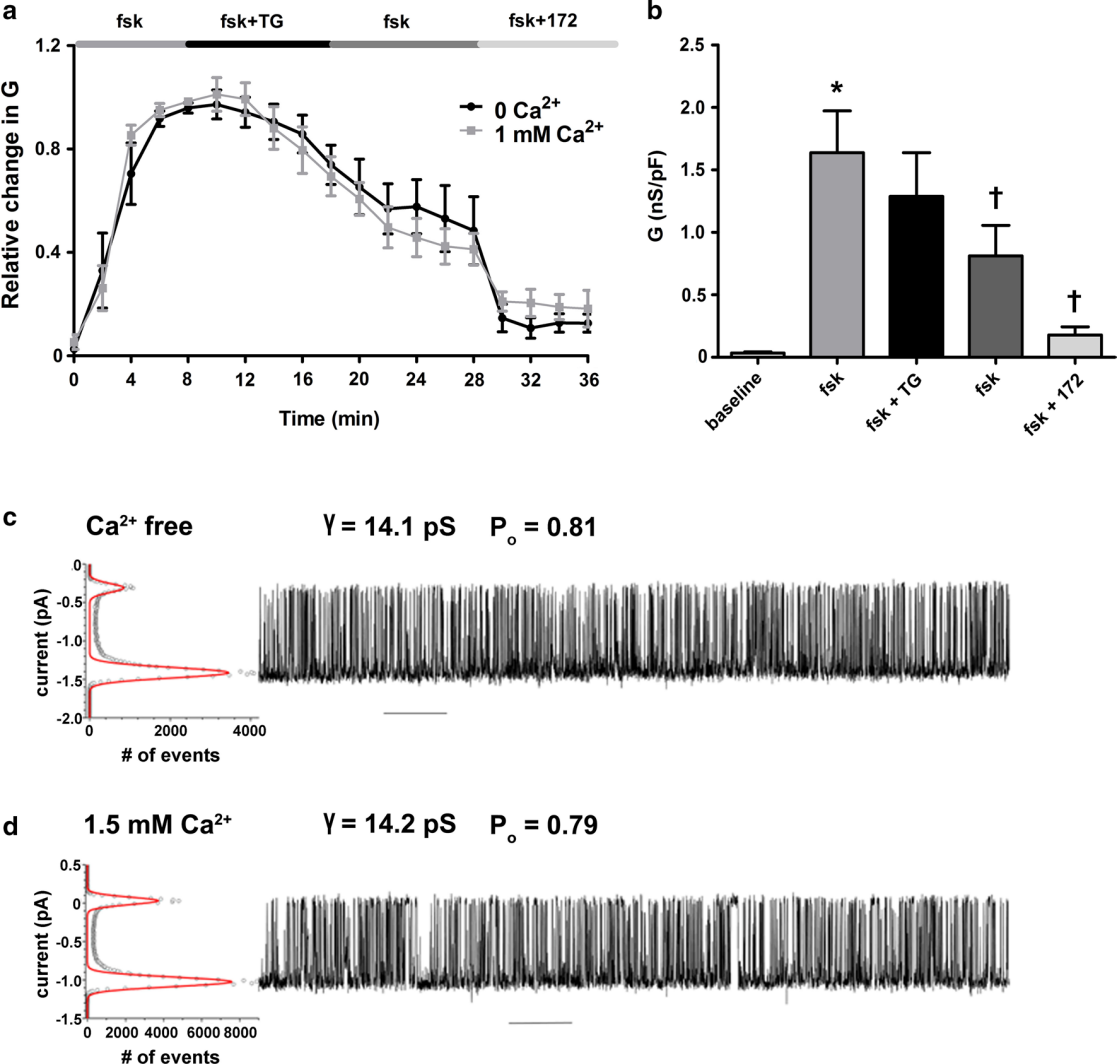
An increase in cytosolic Ca^2+^ from cellular stores causes a decrease in CFTR-mediated conductance without affecting CFTR single channel activity. **a** Changes in current were measured using the fast whole cell configuration of the patch clamp technique. Data are plotted as mean changes in conductance plotted relative to the maximum current reached at + 100 mV when cells were perfused with forskolin (fsk). The black trace represents data from cells exposed to thapsigargin (TG; 200 nM) in nominally Ca^2+^-free conditions. The grey trace represents data from cells exposed to thapsigargin in a bath solution containing 1 mM Ca^2+^. **b** Changes in conductance under the conditions indicated when cells were exposed to thapsigargin in nominally Ca^2+^-free conditions. Conductance was normalised to cell size. Data were analysed from the end of the exposure period to each agonist. Data are mean ± SEM. (*n* = 7) *p < 0.05 when compared to baseline. ^†^*p* < 0.05 when compared to initial forskolin exposure. **c** Single channel recordings (on the right) and all points histogram (on the left) used to calculate single channel conductance and open state probability for CFTR at 37 °C in Ca^2+^-free conditions and **d** after addition of 1.5 mM Ca^2+^ to the cytoplasmic side of the channel. Scale bar represents 10 s interval

We have previously reported that the CS-induced increase in cytoplasmic Ca^2+^ did not require Ca^2+^ influx from the extracellular space [22]. To determine whether the thapsigargin-induced reduction in CFTR-mediated conductance required the presence of extracellular Ca^2+^, experiments were repeated whilst cells were perfused with a nominally Ca^2+^-free solution. Our data showed that the activation of CFTR was no different when cells were exposed to forskolin under nominally Ca^2+^-free conditions (data not shown). Importantly, we found the absence of extracellular Ca^2+^ did not have any effect on the thapsigargin-induced reduction in CFTR-mediated conductance (Fig. 2a; black trace) despite inducing a reduction in the sustained phase of the thapsigargin-induced increase in cytosolic Ca^2+^ (Fig. S1b). Thus, these data suggested that like CS exposure, Ca^2+^ release from cytosolic stores was sufficient to cause a reduction in CFTR at the plasma membrane (Fig. 2b).

Changes in whole cell conductance could be attributed to an effect on the number of channels expressed at the plasma membrane, the single channel conductance, or the open channel probability. To determine whether an increase in cytosolic Ca^2+^ had any direct effect on either the single channel conductance or open probability of CFTR, we measured single channel activity in lipid bilayers expressing purified CFTR. These experiments revealed that an increase in Ca^2+^ on the cytosolic face of CFTR caused no change in single channel conductance. Although not examined in detail, increases in cytosolic Ca^2+^ also had no effect on CFTR gating, since the open probability, and open or closed times looked very similar under low and high Ca^2+^ (Fig. 2c, d). Thus, these data indicated that increases in cytosolic Ca^2+^ influenced the whole cell CFTR conductance by reducing the number of channels at the plasma membrane.

### Increases in cytosolic Ca^2+^ induced by various agonists stimulate CFTR internalisation

**Fig. 3:**
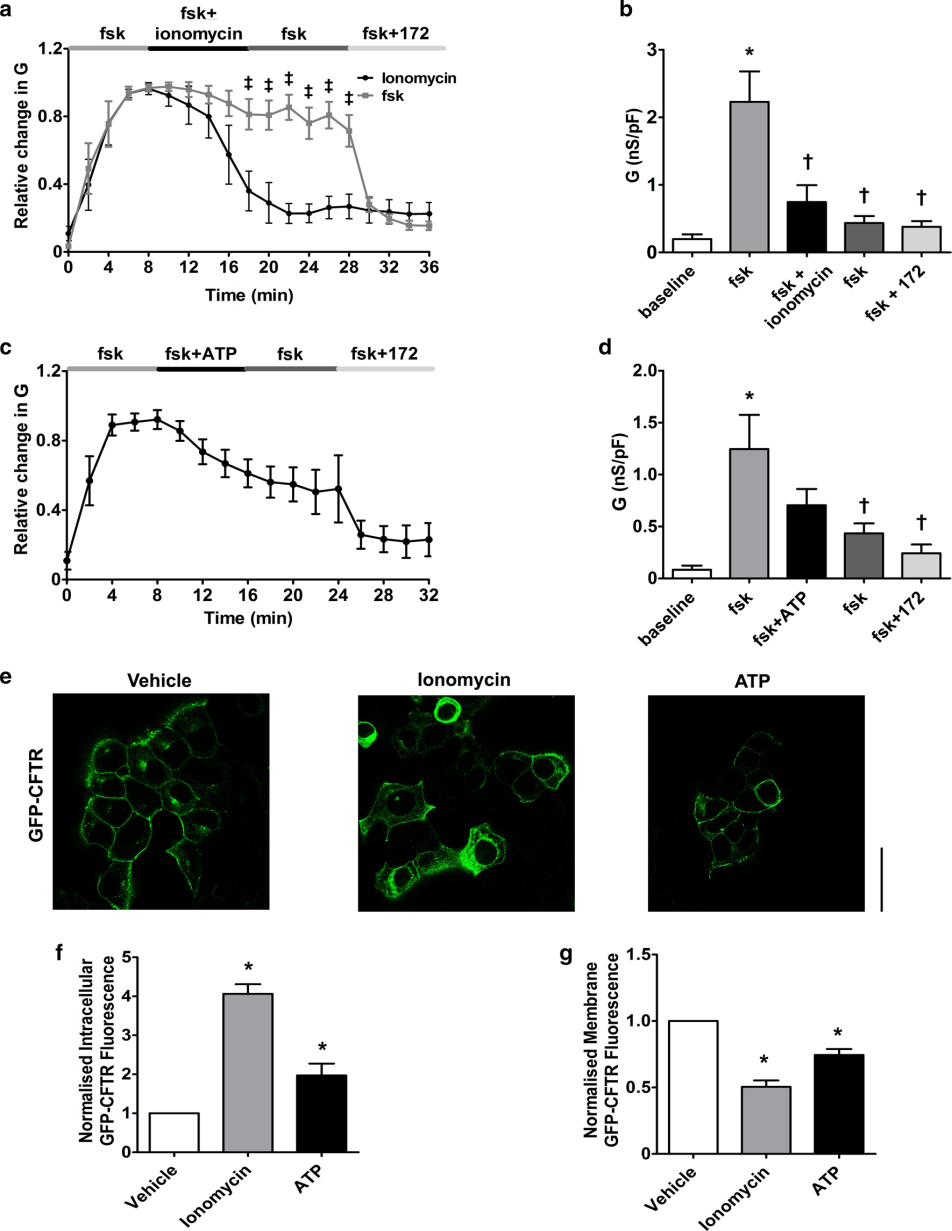
Ca^2+^ agonists cause a loss of CFTR-mediated conductance and internalisation of CFTR (**a**, **c**) HEK293T cells were exposed to forskolin (fsk; 5 µM) followed by a Ca^2+^ agonist and the inhibitor CFTR_inh_-172 (172; 10 µM). Changes in current were measured using the fast whole cell configuration of the patch clamp technique. Data are plotted as mean changes in conductance plotted relative to the maximum current reached at + 100 mV when cells were perfused with forskolin. Cells exposed to either ionomycin (1 µM), forskolin only (taken from Fig. 1c) or ATP (100 µM) as indicated (*n* = 6–8 cells). ^ǂ^*p* < 0.05 when compared to equivalent timepoint in cells exposed to forskolin only. **b**, **d** Changes in conductance under the conditions indicated. Conductance was normalised to cell size. Data was analysed from the end of the exposure period to each agonist. Data are mean ± SEM. *p < 0.05 when compared to baseline. ^†^*p* < 0.05 when compared to initial forskolin exposure. **e** Representative images showing the effect of vehicle, 1 µM ionomycin or 100 µM ATP. Changes in **f** intracellular and **g** membrane fluorescence after 30-min exposure to indicated Ca^2+^ agonist. Data are mean ± SEM (*n* = 210–228 cells from 6 coverslips). Scale bar represents 10 µm. *p < 0.05 compared to vehicle

To further characterize the effect of raising cytosolic Ca^2+^ on CFTR-mediated conductance, we assessed the effect of other Ca^2+^ agonists which induce different spaciotemporal increases in cytosolic Ca^2+^ (Fig. S1). As with earlier experiments, CFTR was first activated by forskolin and then cells exposed to the ionophore, ionomycin (1 µM) for 10 min, with CFTR currents monitored for a further 10 min after the wash out of ionomycin. In comparison to the thapsigargin experiments, exposure to ionomycin caused an increased rate of loss of CFTR-mediated conductance, with only 22.9 ± 7.2% of the forskolin-activated conductance remaining after 20 min exposure (Fig. 3a). Furthermore, ionomycin caused a more complete loss of CFTR-mediated conductance, since no CFTR_inh_-172 sensitive conductance remained at the end of the exposure period (Fig. 3b). Confocal microscopy experiments revealed that ionomycin also caused the movement of CFTR to the intracellular space and the loss of CFTR from the plasma membrane (Fig. 3e–g).

**Fig. 4:**
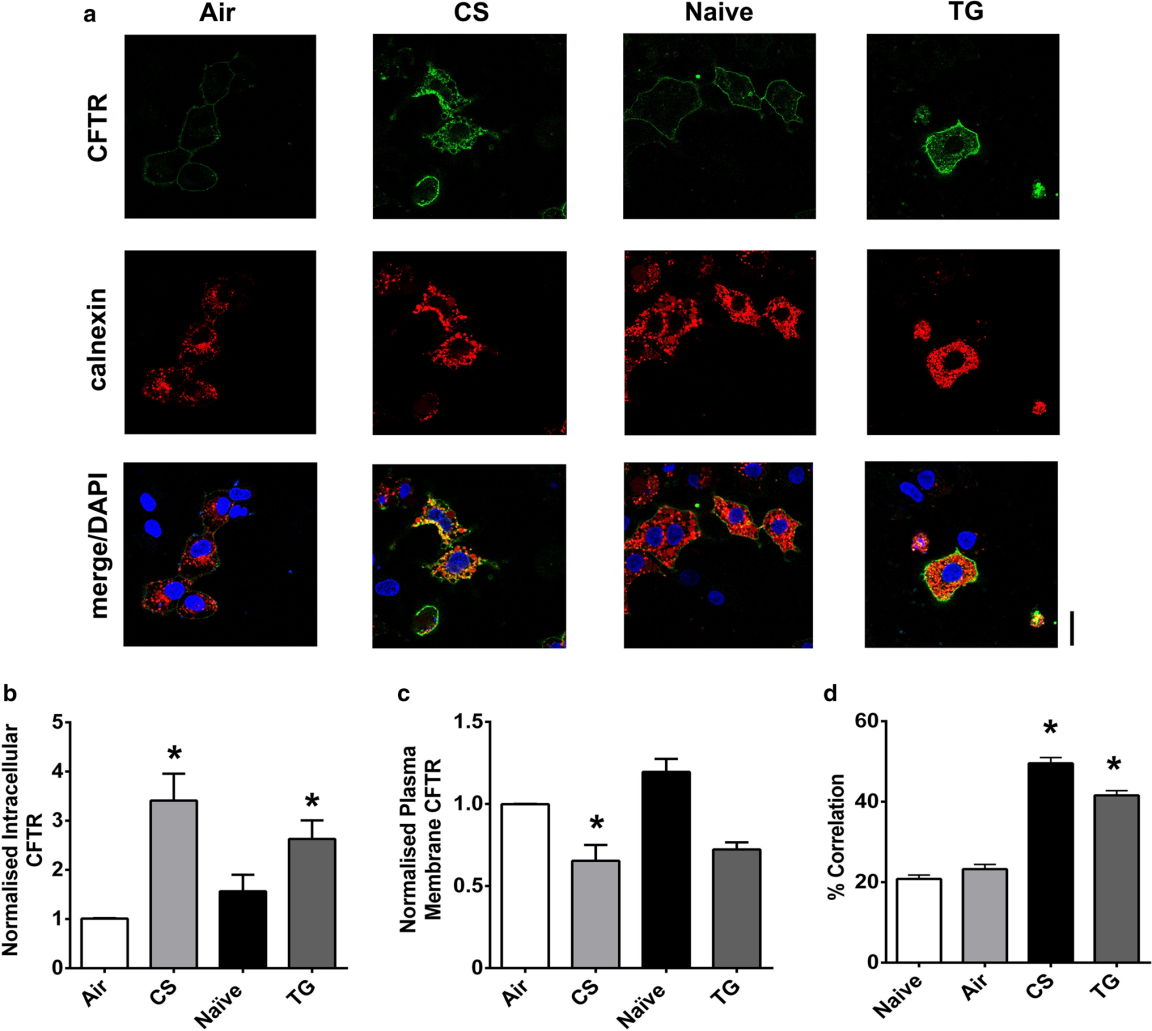
An increase in cytosolic Ca^2+^ causes loss of CFTR from the plasma membrane and internalisation to the endoplasmic reticulum. **a** Representative images showing changes in surface labelled CFTR following exposure to conditions indicated. Changes in **b** intracellular and **c** plasma membrane fluorescence after exposure to indicated agonist. **d** Change in correlation between plasma membrane CFTR and calnexin. Data are mean ± SEM (*n* = 199–323 cells from 6 coverslips). Scale bar represents 25 µm. *p < 0.05 compared to naïve cells

The effect of the physiological agonist, ATP, was also assessed. As with thapsigargin, ATP causes Ca^2+^ release from the ER, mediated through increases in phospholipase C (PLC) [40]. Overall, exposure to ATP (100 µM) caused a significant loss of CFTR-mediated conductance but with some CFTR_inh_-172 sensitive conductance remaining at the end of the exposure period (Fig. 3c, d). Of note, exposure to ATP caused a mixture of responses in CFTR-mediated conductance, with cells showing either no change in conductance, a transient loss in conductance or a reduction in CFTR-mediated conductance. In contrast, the changes in CFTR-mediated conductance induced by thapsigargin and ionomycin were always of the same trend. A similar effect of ATP was seen in confocal imaging experiments, suggesting that even physiological increases in cytosolic Ca^2+^ induce some loss of CFTR from the plasma membrane (Fig. 3e–g). In this regard, we found that both the maximum change, as well as the total increase in cytosolic Ca^2+^ induced by each agonist, strongly correlated with the loss of CFTR-mediated conductance, suggesting that increases in cytosolic Ca^2+^ were clearly associated with loss of CFTR from the plasma membrane (Fig. S1e & f). To further validate our findings in HEK293T cells that increases in Ca^2+^-induced internalisation of CFTR, we measured changes in surface labelled CFTR, using a HA-tag linked to the extracellular domain of CFTR, following exposure to thapsigargin or CS. Our data showed that increases in Ca^2+^ caused a loss of surface labelled CFTR from the plasma membrane. Furthermore, this internalised CFTR co-localised with the endoplasmic reticulum marker, calnexin, suggesting CFTR was being relocated to this organelle (Fig. 4a–c). To provide further support for a loss of cell surface CFTR upon thapsigargin or CS exposure, On-cell Western blot experiments were performed which showed a time-dependent reduction in surface localised CFTR (Fig S2), that followed a similar time course to the GFP fluorescent experiments.

### Increases in cytosolic Ca^2+^ cause internalisation of CFTR in human bronchial epithelial cells

**Fig. 5:**
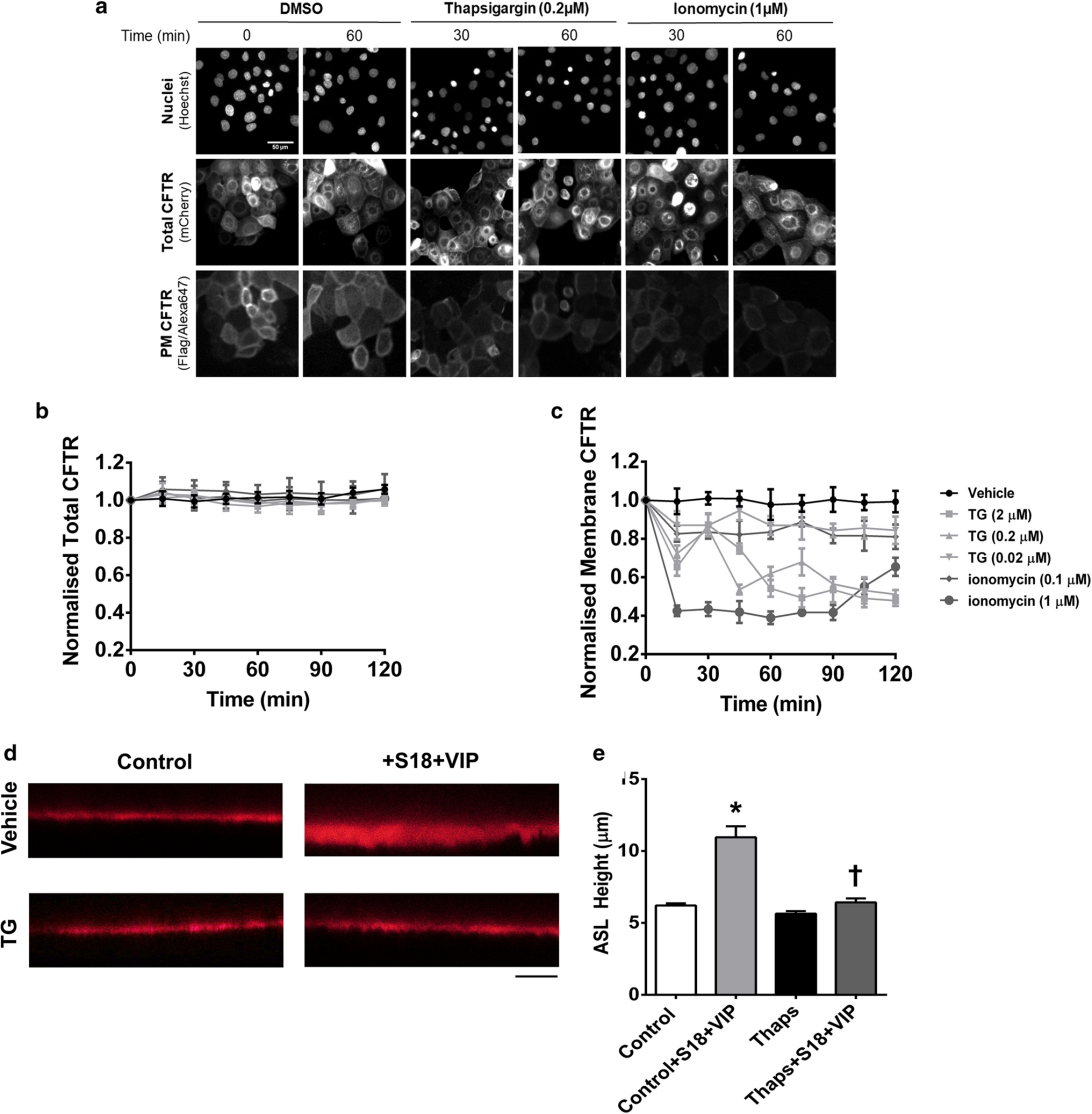
Thapsigargin and ionomycin cause internalisation of CFTR in bronchial epithelial cells. **a** Representative images of CFBE cells expressing the WT-CFTR construct harboring an N-terminal mCherry-tag as a readout for total CFTR and a Flag-tag in the 4th extracellular loop as a readout for plasma membrane CFTR. Cells were treated with vehicle, thapsigargin or ionomycin as indicated. Changes in fluorescence for **b** total and **c** plasma membrane CFTR when exposed to Ca^2+^ agonists as indicated. Data are mean ± SEM (*n* = 4–6). **d** Representative images showing ASL height when exposed to conditions indicated. Scale bar represents 25 µm. **e** Differences in ASL height under conditions indicated. Data are mean ± SEM (*n* = 9 transwells from 3 donors). *p < 0.05 compared to control. ^†^*p* < 0.05 compared to control + S18 + VIP

We next tested whether the observed effect of increases in cytosolic Ca^2+^ could be replicated in epithelial cells, using the CFBE41o^−^ model cell line stably expressing a double tagged CFTR construct that allows assessment of both surface and total CFTR levels in a single cell [29]. Similar to the results in HEK293T cells, treatment with either thapsigargin or ionomycin led to a marked decrease in the steady-state levels of CFTR at the plasma membrane, but did not affect the total amount of CFTR in the cells (Fig. 5a–c). These results indicate that thapsigargin and ionomycin induced internalisation, but not degradation, of CFTR in CFBE cells.

As a further proof that increases in cytosolic Ca^2+^ reduced CFTR function at the plasma membrane, we measured ASL height in primary human bronchial epithelial cells, as a measure of CFTR-dependent Cl^−^ secretion [27]. When cultures were exposed to the CFTR agonist, vasoactive intestinal peptide (VIP), together with the epithelial sodium channel (ENaC) antagonist S18, cultures showed the expected increase in ASL height due to a stimulation of Cl^−^ efflux via CFTR, and inhibition of ENaC by S18 [41]. However, if cultures were first pre-treated with thapsigargin, the VIP/S18 induced increase in ASL height was lost (Fig. 5d, e). These data suggested that loss of CFTR from the plasma membrane correlated with a reduced ASL height which would be predicted to have detrimental effects on airways hydration, in a similar manner to CS.

### An increase in cytosolic Ca^2+^ causes dynamin-dependent internalisation of CFTR that is not dependent on the phosphorylation status of the channel

**Fig. 6:**
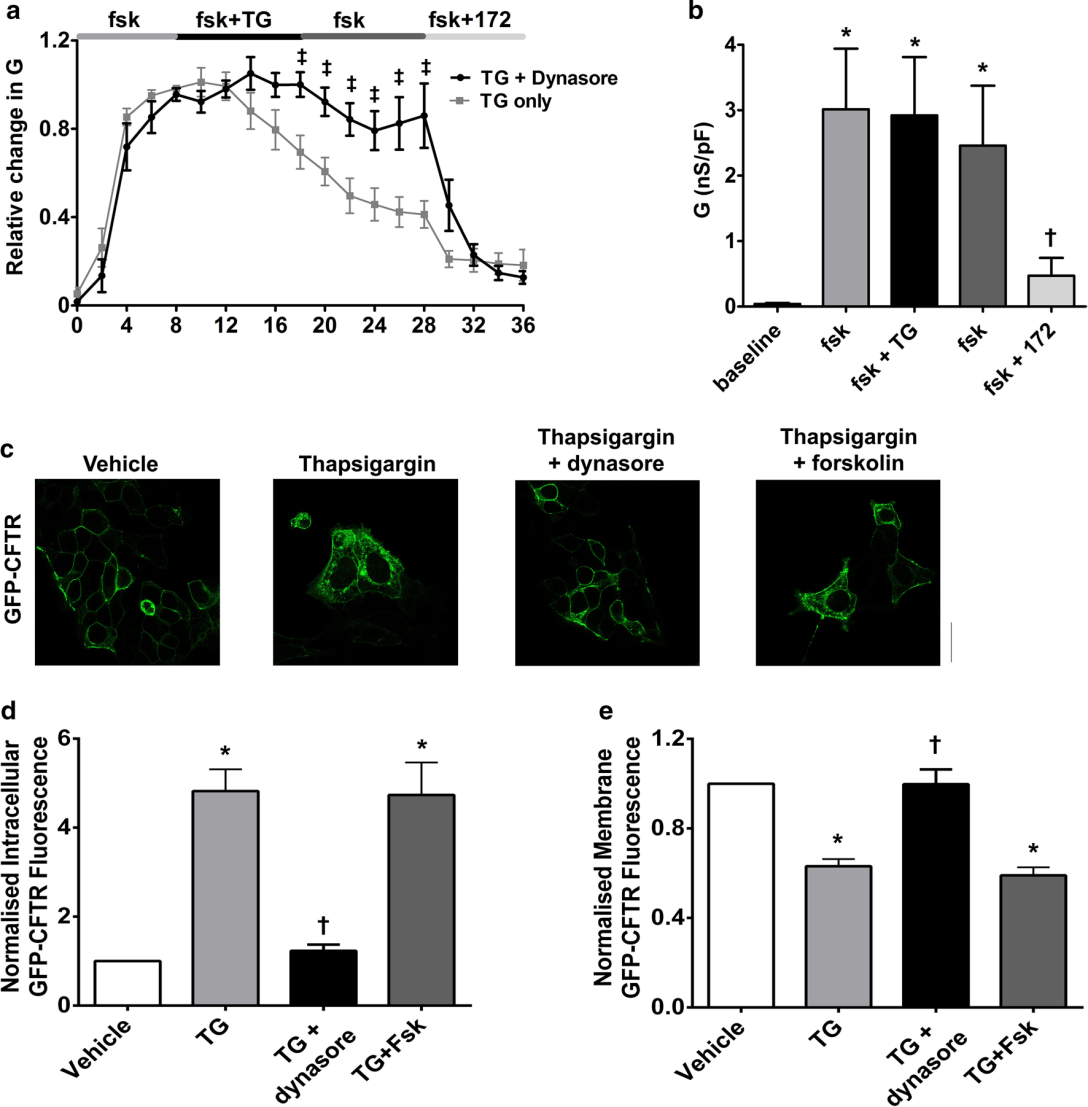
An increase in cytosolic Ca^2+^ causes dynamin-dependent internalisation of CFTR. **a** Cultures were pre-treated with dynasore (80 µM; black trace) for 30–60 min in media at 37 °C and then exposed to thapsigargin (TG; 200 nM) and the inhibitor, CFTR_inh_-172 (172; 10 µM). Changes in current were measured using the fast whole-cell configuration of the patch clamp technique. Data are plotted as mean changes in conductance plotted relative to the maximum current reached at +100 mV when cells were perfused with forskolin (fsk; 5 µM). Grey trace indicates cells that had not been pre-treated with dynasore. **b** Changes in conductance under conditions indicated. Conductance was normalised to cell size. Data were analysed from the end of the exposure period to each agonist. Data are mean ± SEM (*n* = 7 cells). *p < 0.05 when compared to baseline. ^†^*p* < 0.05 when compared to initial forskolin exposure. **c** Representative images showing the effect of vehicle, thapsigargin and dynasore + thapsigargin on CFTR or the effect of vehicle, thapsigargin and thapsigargin + forskolin. Changes in **d** intracellular and **e** membrane fluorescence after 30 min exposure to indicated Ca^2+^ agonist. Data are mean ± SEM (*n* = 199–323 cells from 6 coverslips). Scale bar represents 10 µm. *p < 0.05 compared to vehicle-treated cells ^†^*p* < 0.05 compared to thapsigargin

Under normal conditions, CFTR internalisation is dynamin-dependent [42]. To assess whether an increase in cytosolic Ca^2+^ acted via the same mechanism, HEK293T cells were pre-treated with the dynamin GTPase inhibitor, dynasore (80 µM) for 30–60 min before exposure to thapsigargin. Pre-treatment with dynasore significantly blunted the thapsigargin-induced decrease in CFTR-mediated conductance, consistent with thapsigargin inducing a dynamin-dependent internalisation of CFTR (Fig. 6a, b). To confirm these findings, we used confocal imaging experiments and found that pre-treatment of HEK293T cells with dynasore also prevented the loss of CFTR from the plasma membrane (Fig. 6c). In addition, on-cell Western blot experiments showed co-expression of a dominant negative dynamin mutant, K44A, with HA-CFTR largely blunted the thapsigargin-induced internalisation of CFTR (Fig. S2). These data, therefore, indicated that an increase in cytosolic Ca^2+^ leads to the loss of cell surface CFTR through dynamin-dependent internalisation.

As patch clamp experiments required forskolin pre-treatment to stimulate CFTR channel activity, we also carried out imaging experiments in the presence and absence of forskolin to determine whether phosphorylation via PKA could influence CFTR internalisation. Surprisingly, the phosphorylation state of the channel had no effect on CFTR internalisation or loss of CFTR from the plasma membrane induced by thapsigargin (Fig. 6).

**Fig. 7:**
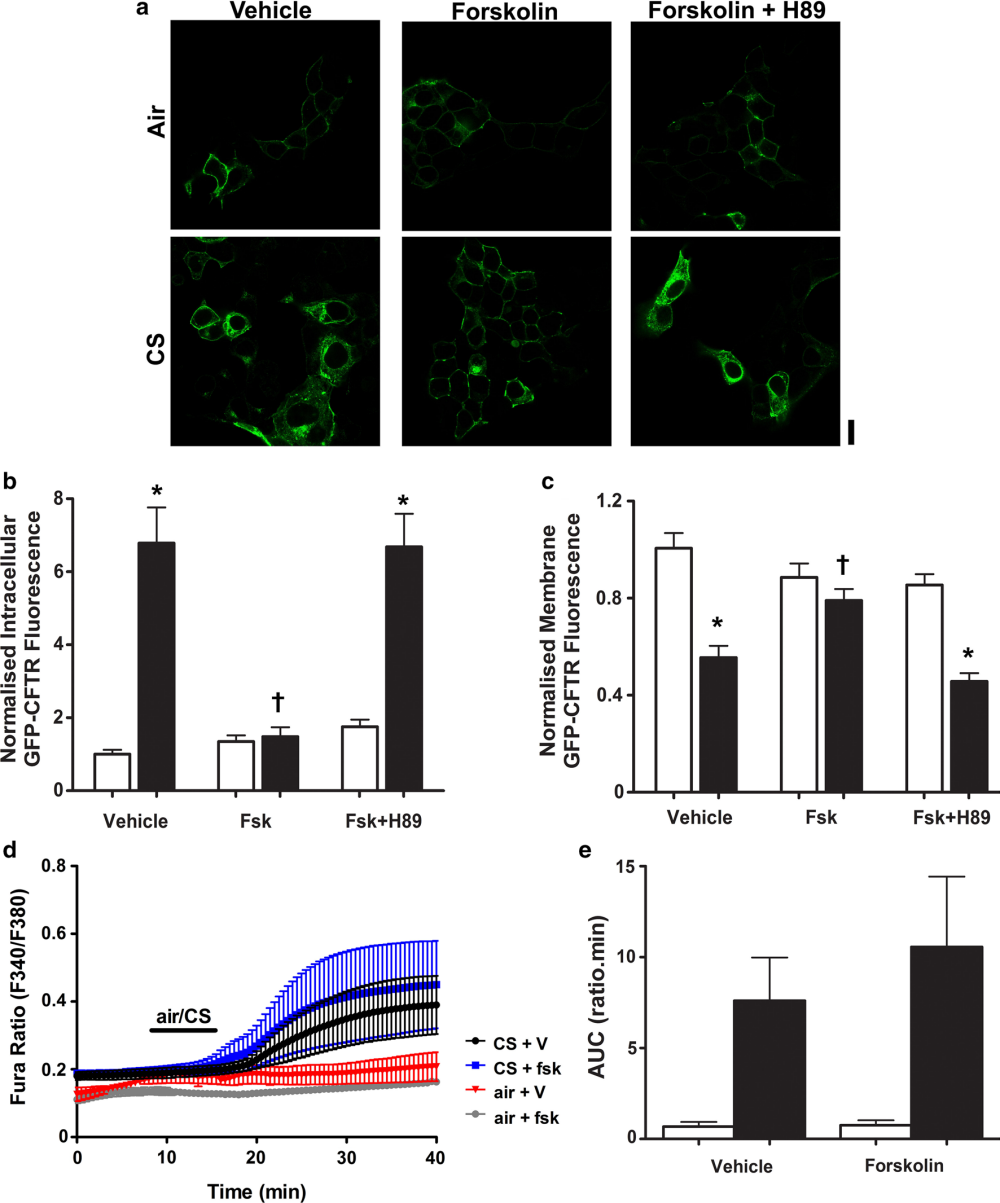
Phosphorylation of CFTR protects against smoke-induced internalisation of the channel. **a** Representative images showing the effect of air or cigarette smoke (CS) exposure after pre-treatment with the either vehicle, forskolin (fsk; 5 µM) or fsk + H89 (0.5 µM). Changes in **b** intracellular and **c** membrane fluorescence under conditions indicated after exposure to air (open bars) or smoke (closed bars) for 30 min. Data are mean ± SEM (*n* = 232–245 cells from 6 coverslips). Scale bar represents 10 µm. *p < 0.05 compared to vehicle. ^†^*p* < 0.05 compared to smoke. **d** Changes in cytosolic Ca^2+^, as indicated by Fura-2 ratio, when cells were pre-treated with either vehicle or forskolin and then exposed to either air or cigarette smoke. **e** Changes in area under the curve (AUC) under conditions indicated. Data are mean ± SEM (*n* = 3–5)

Because our previous experiments did not investigate whether forskolin-induced phosphorylation of CFTR impacted on the effect of CS exposure [22], we conducted additional experiments employing CS. Paradoxically, when forskolin was used to phosphorylate CFTR before exposure to CS, we found that internalisation of the channel was prevented (Fig. 7a–c). To test whether this effect was PKA dependent, cells were also treated with forskolin plus the PKA inhibitor, H89, and then exposed to CS or air. In the presence of H89, forskolin failed to prevent CS-induced CFTR internalisation (Fig. 7a–c), suggesting that this was a PKA-dependent effect. To further understand the different responses to thapsigargin and CS, we tested whether forskolin altered the CS-induced increase in cytosolic Ca^2+^ (Fig. 7d, e). However, we found that forskolin had no effect on the CS-induced Ca^2+^ increase, suggesting that the inhibitory effect of phosphorylation on internalisation was downstream of the CS-induced increase in cytosolic Ca^2+^.

### An increase in cytosolic Ca^2+^ causes activation of calcineurin phosphatase which causes internalisation of CFTR

**Fig. 8:**
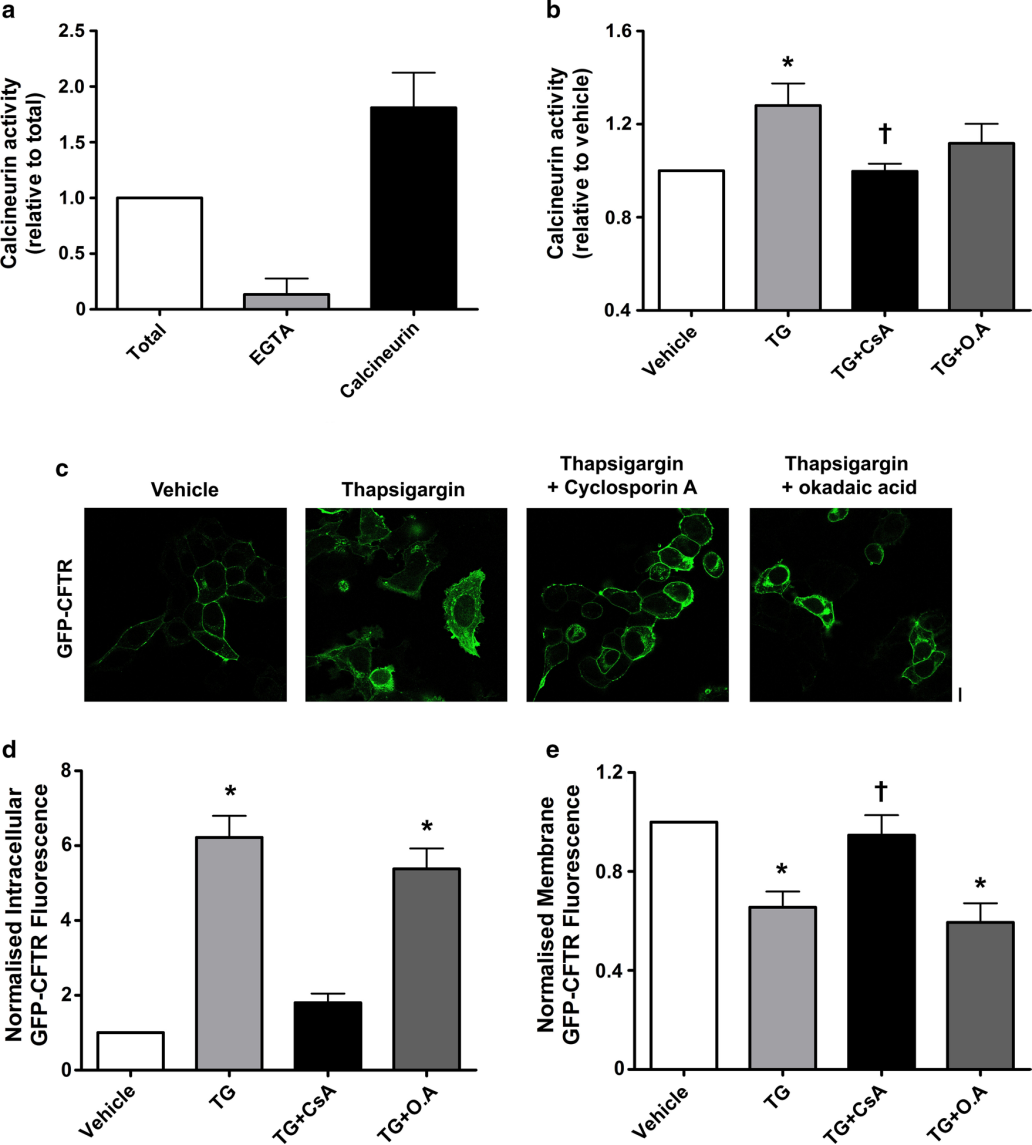
Thapsigargin causes an increase in calcineurin activity which is linked to CFTR internalisation. Calcineurin phosphatase activity was measured using the Enzo Life Sciences calcineurin activity assay. Cells were treated with the conditions indicated and lysed; excess phosphates were then removed and total calcineurin activity assayed. **a** Changes in calcineurin phosphatase activity when treated with  the following: okadaic acid (O.A), EGTA and human recombinant calcineurin. Data have been normalised to total phosphatase activity. **b** Changes in phosphatase activity when cells were exposed to thapsigargin (TG) and various inhibitors; cyclosporin A (CsA). Data have been normalised to vehicle. Data are mean ± SEM (*n* = 5) *p < 0.05 when compared to vehicle. ^†^*p* < 0.05 compared to thapsigargin. **c** Representative images showing the effect of vehicle, thapsigargin (TG; 200 nM), thapsigargin + cyclosporin A (1 µM) and thapsigargin + okadaic acid (10 nM). Changes in **d** intracellular and **e** membrane fluorescence after exposure to conditions indicated for 30 min. Data are mean ± SEM (*n* = 321–363 cells from 6 coverslips). Scale bar represents 10 µm. *p < 0.05 compared to vehicle

As an increase in cytosolic Ca^2+^ is a common step when cells are exposed to either thapsigargin or CS, we investigated the role of the Ca^2+^-dependent phosphatase, calcineurin in CFTR internalisation. To assess whether an increase in cytosolic Ca^2+^ could induce the activation of calcineurin, we measured the activity of this phosphatase after exposure to thapsigargin. Here, we used EGTA (1 mM) as a negative control to inhibit total calcineurin activity, and human recombinant calcineurin (40 U) as a positive control. In total, these controls inhibited or caused a large response in calcineurin activity, respectively, when compared to total cellular calcineurin activity (Fig. 8a). Exposing cells to thapsigargin caused a significant increase in calcineurin phosphatase activity. Importantly, this was prevented by cyclosporin A (1 µM) a specific calcineurin inhibitor, but not by okadaic acid, a PP1 and PP2A inhibitor (10 nM; Fig. 8b).

Following the activation of calcineurin by thapsigargin, we tested whether inhibition of the phosphatase using cyclosporin A would affect CFTR internalisation. Our findings showed that inhibition of calcineurin by cyclosporin A attenuated the thapsigargin-induced internalisation of CFTR (Fig. 8c). In contrast, inhibition of PP1 and PP2A with okadaic acid had no effect on thapsigargin-induced CFTR internalisation (Fig. 8d). Likewise, thapsigargin had no effect on plasma membrane expressed CFTR when cells were pre-treated with cyclosporin A. However, pre-treatment with okadaic acid failed to protect CFTR from thapsigargin-induced internalisation (Fig. 8e).

## Discussion

Tobacco smoke has been shown to cause internalisation of CFTR through a smoke-induced increase in cytosolic Ca^2+^ [22]. However, the underlying mechanism is poorly understood. Here, we have demonstrated that an increase in cytosolic Ca^2+^, regardless of the source or mechanism, caused a decrease in CFTR-mediated conductance via internalisation of the channel. Similarly, Bargon and colleagues (1992) found that exposure of HT-29 cells to agents that increased the intracellular concentration of divalent cations, including Ca^2+^, caused a reduction in CFTR mRNA and protein in a time- and dose-dependent manner [43], validating our data showing that increases in cytosolic Ca^2+^ alone are able to reduce CFTR activity.

Experiments testing the effect of thapsigargin on CFTR activity under nominally Ca^2+^-free conditions suggested that extracellular Ca^2+^ was not necessary for the loss of CFTR-mediated conductance, and, therefore, store-operated Ca^2+^ entry was not required. Similarly, Rasmussen and colleagues found that if cells were exposed to CS in the absence of extracellular Ca^2+^, the increase in cytosolic Ca^2+^ was similar to that seen when Ca^2+^ was present [22]. These data suggest a transient increase in Ca^2+^, derived from internal stores, was sufficient to cause CFTR internalisation.

Single channel experiments showed that an increase in cytosolic Ca^2+^ had no effect on either the conductance or open probability of purified CFTR studied in lipid bilayers. These findings are consistent to those reported by Clunes et al. who carried out similar experiments in bilayers that had been exposed to CS [4]. The data support the evidence that exposure to CS, or to an agonist that increases cytosolic Ca^2+^, causes a reduction in CFTR function via an effect on the number of channels present on the membrane and not the gating of the channel. However, Bozoky and colleagues found that an increase in Ca^2+^ in the presence of forskolin caused a reduction in CFTR activity [43], when measured in excised patches. The reason for the difference in response to raised Ca^2+^ in excised patches versus purified CFTR in an artificial bilayer is not known. One possibility is that in the excised patch experiments, there was a Ca^2+^-dependent phosphatase present which, if activated, could decrease CFTR activity via dephosphorylation. This protein would be lost during purification for the bilayer experiments reported here.

To test the effect of a physiological agonist on CFTR-mediated conductance, cells were exposed to ATP. Usually released in response to the mechanical stresses seen during breathing, ATP causes the release of Ca^2+^ from ER stores [44]. A proportion of cells exposed to ATP showed a transient loss of CFTR-mediated conductance. Accordingly, stimulation of the P2Y_2_ receptor causes activation of the G_q_ pathway and the activation of PKC via this pathway results in the phosphorylation of CFTR which potentiates the effect of PKA [44]. Furthermore, ATP is broken down to adenosine by ectonucleotidases, stimulating CFTR in a autocrine/paracrine manner through the action of adenosine on the A_2B_ receptor [45]. Likewise, the activation of the A_2B_ receptor has been linked to cAMP accumulation via G_s_ [46, 47]. Therefore, it could be that the increase in cytosolic Ca^2+^ induced by ATP caused a loss of CFTR channels at the cell surface, but the recovery was due to one of the effects listed above. Most cells tested in our experiments showed some decrease in conductance, either as an initial decrease followed by a plateau or a gradual decline in conductance. This suggests that the increase in cytosolic Ca^2+^ caused by ATP may have overwhelmed the ability of the various mechanisms listed above to prevent a loss in conductance.

Thapsigargin and ionomycin typically cause supraphysiological increases in cytosolic Ca^2+^. However, ATP likely caused physiological increases in Ca^2+^. In our study, the dosage used may account for the proportion of cells which showed a reduction in CFTR activity. However, it has been suggested that low levels of increases in cytosolic Ca^2+^ can stimulate Ca^2+^-dependent adenylyl cyclase and PKA phosphorylation of CFTR, suggesting that physiological increases in cytosolic Ca^2+^ are likely to cause an increase in CFTR activity [47]. Further to these mechanisms, Billet and Hanrahan have suggested that CFTR itself can act as a CaCC [48]. Indeed, Billet et al. showed that Ca^2+^ activated tyrosine kinases, such as Pyk2, can activate CFTR through tyrosine phosphorylation of the channel and activation of the Pyk2/Src complex [49].

Our data showed that the Ca^2+^-induced loss of CFTR from the plasma membrane and subsequent internalisation could be replicated in either a bronchial epithelial cell line (CFBE41o^−^) or in fully differentiated primary human bronchial cells, where CFTR signalling is highly compartmentalised. Furthermore, the loss of CFTR from primary cultures was associated with a failure of these bronchial epithelial cells to respond to physiological agonist, as measured by a change in ASL height. As an abnormal ASL height has been linked to an impairment of mucociliary clearance and an increased prevalence of bacterial infections [50], our data provide an insight into how an increase in cytosolic Ca^2+^ could result in detrimental effects on the airway microenvironment via changes in CFTR expression at the plasma membrane.

The dynamin GTPase inhibitor, dynasore [42], prevented a Ca^2+^-induced decrease in conductance, suggesting that the Ca^2+^-dependent decrease in whole cell conductance was due to a dynamin-dependent endocytosis of CFTR. These data were supported by expression of a dominant negative dynamin mutant, which blunted the thapsigargin-induced CFTR internalisation. Dynamin is the primary protein involved in scission of clathrin-coated pits from the cell membrane and dynasore inhibits the GTPase activity of dynamin by preventing the hydrolysis of GTP [51]. Previous studies have shown that dynasore is able to prevent the endocytosis of CFTR under resting conditions, suggesting that the initial mechanism by which CFTR is internalised, following an increase in cytosolic Ca^2+^, resembles that of the normal trafficking process [52]. Interestingly, cytosolic Ca^2+^ increases have been linked to regulating the balance between endocytosis and exocytosis. In synaptosomes, Marks and McMahon showed that an increase in Ca^2+^ was able to cause endocytosis at concentrations lower than that needed for exocytosis [53]. Furthermore, in nerve terminals, an increase in cytosolic Ca^2+^, and binding of Ca^2+^ to calmodulin, has been shown to be linked to the initiation of endocytosis [54].

As we have previously reported, we found that acute exposure to the volatile phase of CS caused internalisation of CFTR [4, 22]. Wong and colleagues have recently found cigarette smoke extract (CSE) caused a reactive oxygen species-dependent increase in CFTR activity. This increase in activity was followed by a decline after prolonged exposure to CSE [55]. However, the researchers used CSE which captures a different phase of smoke than our preparation. Nevertheless, we have recently linked various components of CS, such as nicotine, to an increase in cytosolic Ca^2+^ [56]. Indeed, alterations in nicotinic acetylcholine receptor activation linked to CS have been linked to CFTR dysfunction [57]. Thus, our findings provide a link between exposure of cultures to CS, the subsequent increase in cytosolic Ca^2+^ and loss of CFTR.

Our study revealed that forskolin pre-treatment had no effect on Ca^2+^-induced internalisation of CFTR; however, it did prevent CS-induced loss of CFTR. Whilst no other studies have investigated the effect of an increase in cytosolic Ca^2+^ on CFTR internalisation, others have found that forskolin prevented endocytosis of CFTR in pancreatic and colonic cells [58, 59]. Therefore, it seems that when cells are exposed to an increase in cytosolic Ca^2+^, phosphorylation via PKA is not able to protect CFTR. Arguably, CS exposure would cause numerous effects within a cell. It is possible that besides the increase in cytosolic Ca^2+^, another consequence of CS exposure is responsible for this discrepancy between the effect of CS and an increase in cytosolic Ca^2+^; for instance, CS contains constituents which could activate cell surface or intracellular signalling pathways that are not directly linked to changes in cytosolic calcium [60], but which could also influence CFTR surface expression and dependence on PKA phosphorylation. Since CS is a very complex mixture of chemicals [61, 62] further work will be required to fully understand this discrepancy.

We showed that an increase in Ca^2+^ caused activation of the Ca^2+-^dependent phosphatase, calcineurin, which is known to regulate CFTR activity. Fischer et al. [13] found that calcineurin inhibited the activation of CFTR by either PKA or PKC. However, there has been some uncertainty regarding the regulation of CFTR by calcineurin, with the suggestion that the effect of calcineurin may depend on the cellular location of the phosphatase [13]. Thus, an increase in cytosolic Ca^2+^ may cause the movement of calcineurin to bring it into close proximity of CFTR to enable dephosphorylation of the channel. In this regard, Lai et al. [63] found that in extracts from rat brain, there was a Ca^2+^-dependent interaction between calcineurin and dynamin 1. Further, an increase in cytosolic Ca^2+^ was linked to the dephosphorylation of dynamin, resulting in the translocation of the calcineurin–dynamin complex to a cluster of proteins involved in clathrin-mediated endocytosis [63]. Given that inhibition of dynamin via dynasore was found to inhibit CFTR internalisation, these data from Lai and colleagues provide a functional link between the data showing a role for calcineurin and dynamin in Ca^2+^-induced internalisation of CFTR. Our data suggested that inhibition of calcineurin may be beneficial in cells exposed to CS, as cyclosporin A and analogues such as tacrolimus are currently in clinical use as immunosuppressants [64]. However, the use of immunosuppressants in patients may be of limited use as these agents would undoubtedly cause an increased likelihood of patients becoming susceptible to infection. Thus, our data open the possibility of focusing on the development of CFTR modulators that stabilise CFTR at the cell surface, or the phosphorylation status of CFTR, as therapeutic targets. Likewise, the CFTR potentiator, ivacaftor (VX-770), used in the treatment of CF, may have promise in the treatment of COPD. Indeed, Raju and colleagues have found that treatment of human bronchial epithelial cultures with ivacaftor prevented any smoke-related effects on CFTR [65].

In conclusion, we have shown that agonists that promote a rise in cytosolic Ca^2+^ are able to modulate the cell surface expression of CFTR at the plasma membrane, through a dynamin- and calcineurin-dependent mechanism. These findings help provide a better mechanistic understanding of how changes in cytosolic Ca^2+^ negatively impact CFTR function and help explain the action of CS on CFTR function. Given that CS is a major cause of lung disease and that loss of CFTR expression induced by CS is Ca^2+^-dependent, our data may help in the development of new strategies to minimise the deleterious effect of CS on CFTR function. Together, we hypothesise that these strategies would prevent dehydration of the ASL and consequently could be beneficial to lung health by offsetting the CS-induced loss of CFTR.

## Electronic supplementary material

Below is the link to the electronic supplementary material.

**Fig. S1 Spatiotemporal changes in cytosolic Ca**^**2+**^**induced by agonists used in this study** Cultures were exposed to forskolin (fsk; 5 µM) followed by a Ca^2+^ agonist in the presence of forskolin. The agonist was subsequently washed out of the perfusate and cells were exposed to CFTR_inh_-172 (172; 10 µM). Mean changes in cytosolic Ca^2+^, as indicted by changes in Fura-2 AM ratio, induced by thapsigargin (TG; 200 nM) in a (**a**) bath solution containing 1 mM Ca^2+^ or (**b**) nominally Ca^2+^-free solution. Changes in cytosolic Ca^2+^ induced by (**c**) ionomycin (1 µM) and (**d**) ATP (100 µM). Correlation between changes in cytosolic Ca^2+^ and inhibition of CFTR-mediated conductance determined by the average rate of inhibition of CFTR-mediated conductance and (**e**) maximum change in Fura-2 ratio or (**f**) area under the curve (AUC) induced by Ca^2+^ agonists. Data are mean ± SEM (n = 3 independent experiments). (JPEG 1548 kb)

**Fig. S2 K44A Dynamin prevents a thapsigargin-induced CFTR internalisation.** Changes in plasma membrane localised HA-CFTR following exposure to thapsigargin (TG) for times indicated with, or without, co-transfection with the dominant negative dynamin mutant. K44A. Data are mean ± SEM (n = 34-44 cells from 3 independent experiments). * = p < 0.05 compared to t = 0 † = p < 0.05 compared to thapsigargin at 60 min. (JPEG 1082 kb)

## Abbreviations

CFTR: Cystic fibrosis transmembrane conductance regulator
CS: Cigarette smoke
CF: Cystic fibrosis
COPD: Chronic obstructive pulmonary disease
CB: Chronic bronchitis
NBD: Nucleotide binding domain
PP: Protein phosphatase
ER: Endoplasmic reticulum
ASL: Airway surface liquid
HEK: Human embryonic kidney
DMEM: Dulbecco’s modified Eagle’s medium
ENaC: Epithelial sodium channel
FBS: Foetal bovine serum
HBEC: Human bronchial epithelial cell
*I*–*V*: Current–voltage
AUC: Area under curve
SERCA: Sarcoendoplasmic reticulum Ca^2+^ ATPase
PLC: Phospholipase C
CSE: Cigarette smoke extract
VIP: Vasoactive intestinal peptide
