# Supplementary figures and images for: Increases in cytosolic Ca2+ induce dynamin- and calcineurin-dependent internalisation of CFTR
Source: Cell Mol Life Sci. 2018 Dec 13;76(5):977–94. doi: 10.1007/s00018-018-2989-3 (PMC6394554; doi:10.1007/s00018-018-2989-3)

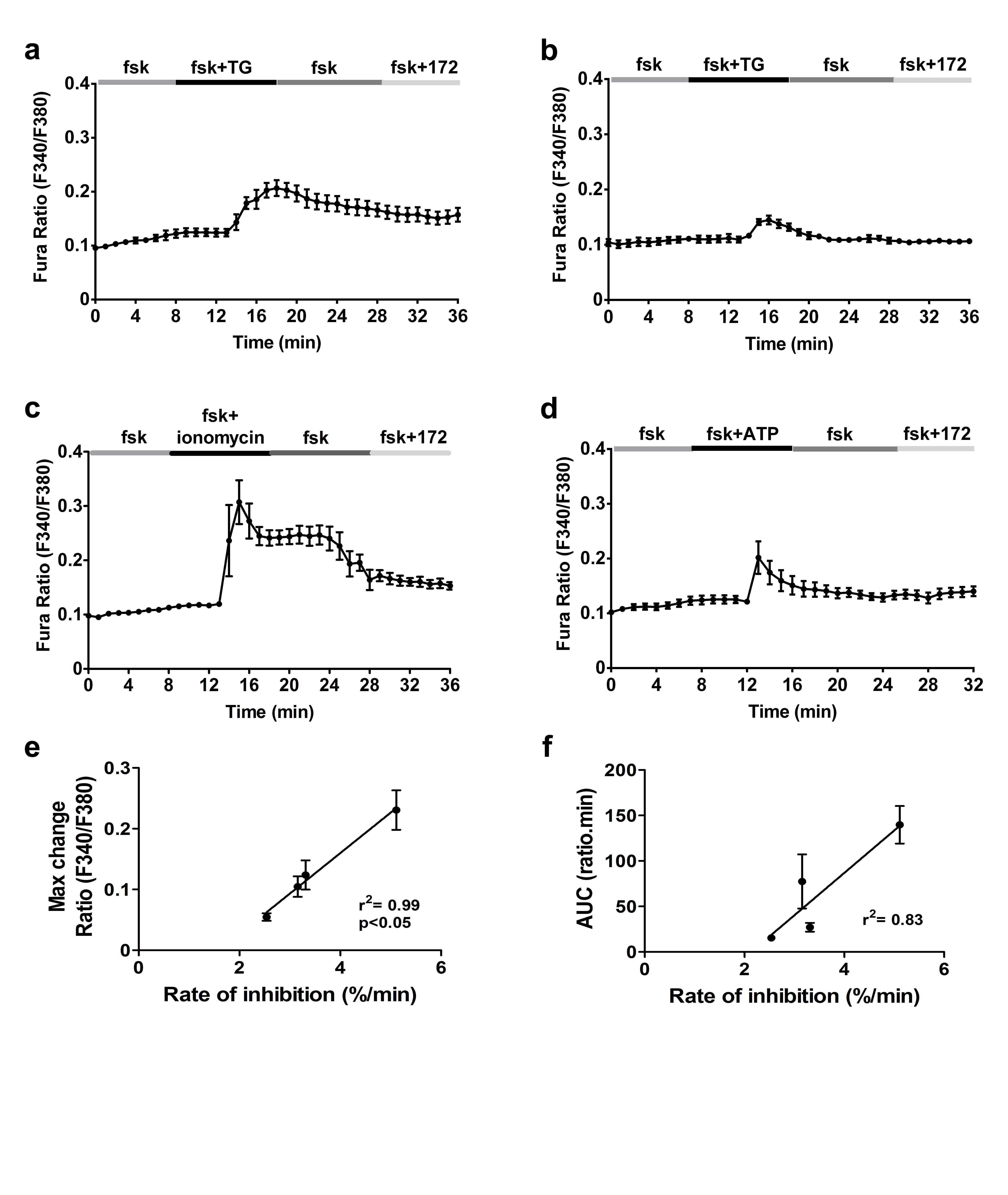

Supplement: Supplementary file 1 — Fig. S1 Spatiotemporal changes in cytosolic Ca2+induced by agonists used in this study Cultures were exposed to forskolin (fsk; 5 µM) followed by a Ca2+ agonist in the presence of forskolin. The agonist was subsequently washed out of the perfusate and cells were exposed to CFTRinh-172 (172; 10 µM). Mean changes in cytosolic Ca2+, as indicted by changes in Fura-2 AM ratio, induced by thapsigargin (TG; 200 nM) in a (a) bath solution containing 1 mM Ca2+ or (b) nominally Ca2+-free solution. Changes in cytosolic Ca2+ induced by (c) ionomycin (1 µM) and (d) ATP (100 µM). Correlation between changes in cytosolic Ca2+ and inhibition of CFTR-mediated conductance determined by the average rate of inhibition of CFTR-mediated conductance and (e) maximum change in Fura-2 ratio or (f) area under the curve (AUC) induced by Ca2+ agonists. Data are mean ± SEM (n = 3 independent experiments). (JPEG 1548 kb) [file 18_2018_2989_MOESM1_ESM.jpg]

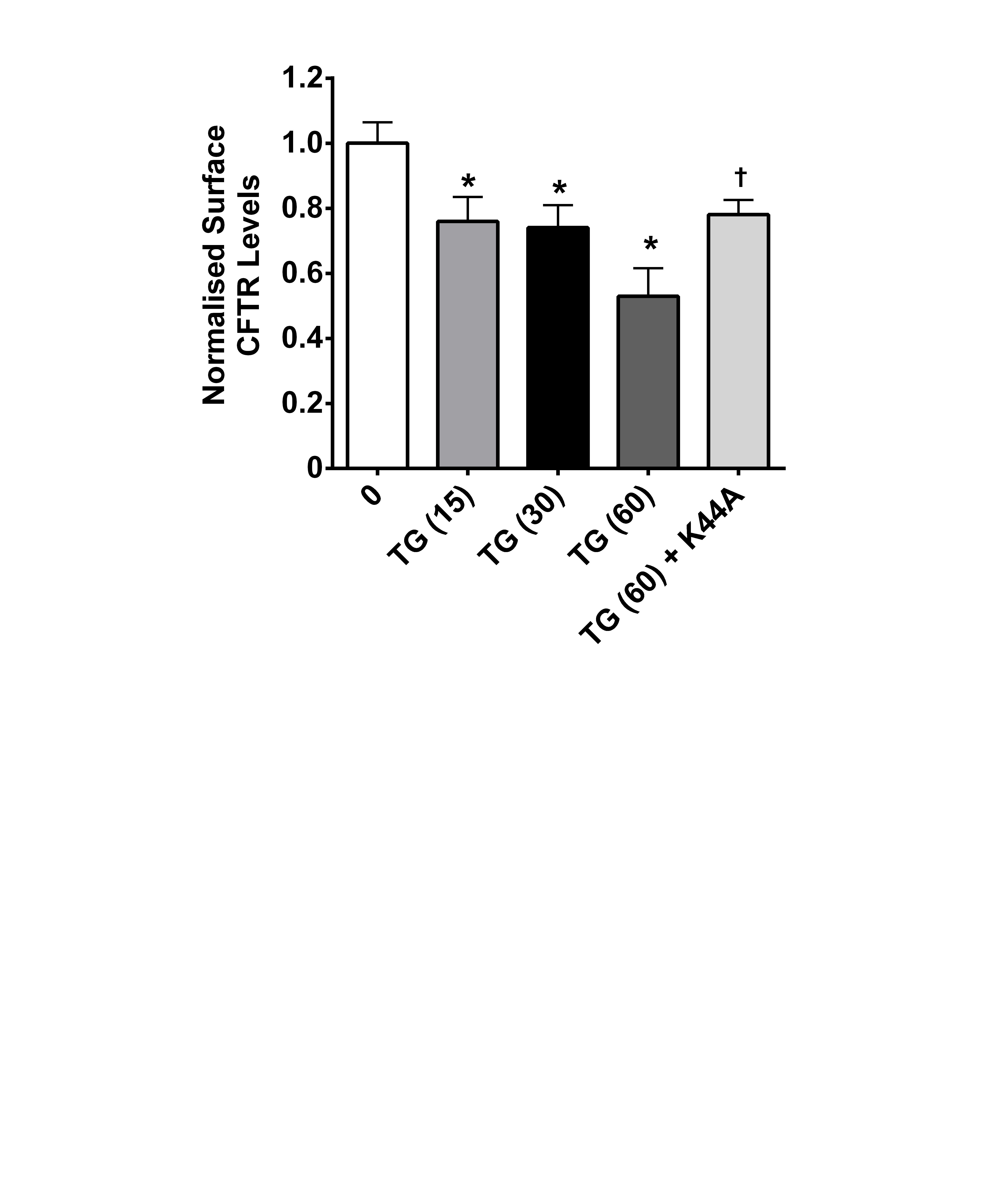

Supplement: Supplementary file 2 — Fig. S2 K44A Dynamin prevents a thapsigargin-induced CFTR internalisation. Changes in plasma membrane localised HA-CFTR following exposure to thapsigargin (TG) for times indicated with, or without, co-transfection with the dominant negative dynamin mutant. K44A. Data are mean ± SEM (n = 34-44 cells from 3 independent experiments). * = p < 0.05 compared to t = 0 † = p < 0.05 compared to thapsigargin at 60 min. (JPEG 1082 kb) [file 18_2018_2989_MOESM2_ESM.jpg]
